# Erector spinae plane block versus caudal epidural block in pediatric surgery: a systematic review and meta-analysis of randomized clinical trials

**DOI:** 10.1016/j.bjane.2025.844640

**Published:** 2025-05-16

**Authors:** Barbara Bombassaro Masiero, Deivyd Cavalcante, Fatemeh Akbarpoor, Capela António Dicazeco Pascoal, Lubna Al-Sharif, Fellipe Feijó Halfeld, Lucas Cael Azevedo Ramos Bendaham, Patricia Viana, Jesslyn N. Haryianto, Maria Luiza de Souza Rasia, Mariana Copetti de Almeida Cunha, Ana Djulia Tesche, Júlia Caletti Roth de Oliveira, Rafael Arsky Lombardi

**Affiliations:** a Pontifícia Universidade Católica do Rio Grande do Sul, Porto Alegre, RS, Brazil; b Universidade Federal do Maranhão, São Luís, MA, Brazil; c Mohammed bin Rashid University of Medicine and Health Sciences, Dubai, UAE; d Faculdade de Medicina da Universidade Agostinho Neto, Luanda, Angola; e An-Najah National University, Nablus, Palestine; f Universidade Federal do Rio de Janeiro, Rio de Janeiro, RJ, Brazil; g Universidade Federal de Roraima, Boa Vista, RR, Brazil; h Universidade do Extremo Sul Catarinense, Criciúma, SC, Brazil; i Pelita Harapan University, Indonesia; j Universidade de Caxias do Sul, Caxias do Sul, RS, Brazil; k University of Nebraska, Lincoln, Kansas, United States of America

**Keywords:** Analgesics, Anesthesia, caudal, Nerve block, Pain measurement, Pediatrics, Postoperative nausea and vomiting, Urinary retention

## Abstract

**Background:**

Caudal Epidural Block (CEB) is a well-established regional anesthesia technique for abdominal and sub-abdominal pediatric surgeries. However, it has a short duration, often leading to additional analgesic administration. Erector Spinae Plane Block (ESPB), for instance, is an emerging technique that, like CEB, provides analgesic effect to a specific dermatome of the body during surgery and in the postoperative period. Therefore, we performed this systematic review with meta-analysis to compare both techniques.

**Methods:**

We searched PubMed, Embase and Cochrane Central for Randomized Controlled Trials (RCTs) comparing ESPB versus CEB in pediatric patients undergoing abdominal and sub-abdominal surgeries. The primary outcome was the time to first analgesic request. Secondary outcomes were I) FLACC score; II) Postoperative nausea and vomiting, and III) Urinary retention.

**Results:**

Nine randomized controlled trials encompassing 507 patients were included in this analysis (1‒9). The patients were predominantly male and under 10 years of age. There was an equal distribution between the two groups regarding the number of patients and patients’ baseline characteristics. The main results were: time to first analgesic request (MD = 3.71; 95% CI: -1.88–9.29; *I^2^* = 99%; p = 0.19); FLACC scores at 2 hours (MD = 0.15; 95% CI: -0.30–0.59; *I^2^* = 0%; p = 0.52); FLACC scores at 24 hours (MD = -0.17; 95% CI: -0.39–0.05; *I^2^* = 41%; *I^2^* = 41%; p = 0.13); urinary retention events (RR = 0.12; 95% CI: 0.02–0.94; *I^2^* = 0%; p = 0.04); and Postoperative Nausea and Vomiting (PONV) which was null in both groups in three studies. However, it is important to clarify that some limitations were identified, such as significant heterogeneity in the following outcomes: time to first analgesic request and FLACC score at 24h, possibly due to different age groups, different types of surgeries, different background analgesia administration, and a relatively small sample size. As for the risk of bias, two studies were found to have some concerns in “bias due to deviations from intended interventions” (8,9).

**Conclusion:**

Our findings suggest that the administration of ESPB did not statistically differ from CEB regarding the time to first analgesic request. FLACC scores also did not show a statistically significant difference between groups. The ESPB group, however, experienced minor urinary retention events compared to the CEB group.

**Quality of evidence:**

According to the GRADE assessment, all outcomes evaluated in this study were classified as high-quality evidence. Quality assessment is detailed in Supplementary Table 1.

## Introduction

Abdominal and sub-abdominal surgeries are often associated with high postoperative pain and usually require long-term analgesia.^1^ To mitigate these events, regional anesthesia is often combined with general anesthesia. In the pediatric population, Caudal Epidural Block (CEB) is a low-cost, simple, and well-established technique.^1^ However, it also has a short duration, often requiring additional analgesics to control pain.^2^

Erector Spinae Plane Block (ESPB) is an emerging regional anesthesia technique for pediatric patients. It has attracted the attention of anesthesiologists and surgeons due to its effective pain management and favorable safety profile.^3^ Previous studies reported adequate analgesia and a low patient complication rate.^4^ ESPB spreads both cranially and caudally, providing extensive dermatomal coverage from the thoracic to lumbar regions, effectively blocking somatic pain from the muscles and skin as well as visceral pain from the thoracic and abdominal organs.^1^

Effective pain management in pediatric surgery is critical, as children are particularly vulnerable to physiological stress responses ‒ such as elevated heart rate and blood pressure ‒ due to their immature nervous and immune systems. These responses can impede recovery and increase the risk of postoperative complications.^5^^,^^6^ Inadequate analgesia has also been linked to lasting psychological effects, including anxiety, post-traumatic stress disorder, and sensitization to future medical procedures.^7^^,^^8^

In contrast, well-managed perioperative pain is associated with faster recovery, fewer complications, and a reduced risk of chronic pain development.^9^^,^^10^ This meta-analysis systematically approaches this question through the comparison of the analgesic efficacy and safety of CEB versus ESPB in pediatric patients undergoing abdominal and sub-abdominal surgeries.

## Methods

This systematic review with meta-analysis was registered in the international prospective register of systematic reviews and clinical trials (PROSPERO) under protocol CRD42024569890, intending to certify transparency and reduce bias risk. This study was designed following the Cochrane Collaboration Handbook for Systematic Review of Interventions and the Preferred Reporting Items for Systematic Reviews and Meta-Analysis (PRISMA) Statement Guidelines.^11^

### Eligibility criteria

Studies were selected for inclusion in this meta-analysis based on the following criteria: I) Pediatric patients under 15-years-old; II) Studies that compared ESPB versus CEB; and III) Randomized controlled trials. We included only original, full-text, peer-reviewed articles published in English to reduce language bias.^12^ Exclusion criteria were: I) Patients over 15-years-old; II) Observational studies; III) Clinical trials ongoing; IV) Conference abstracts; V) Failed nerve blocks; and VI) Articles not published in English.

### Search strategy and data extraction

Pubmed, Embase and Cochrane Central Register of Controlled Trials databases were systematically searched by two authors (B.B.M. and L.A.S.) from July 3^rd^, 2024 to July 18^th^, 2024, to identify studies meeting all the inclusion criteria. The search strategy used was: “(pediatric OR peadiatric OR paediatric OR children OR infant OR infants OR child OR adolescent OR adolescents) AND (“erector spinae plane block” OR “ESP block” OR ESPB OR “erector spinae plane nerve block” OR “erector spinae muscle block”) AND (“caudal block” OR “caudal epidural nerve block” OR “caudal epidural block” OR “caudal anesthesia” OR “epidural” OR “caudal anaesthesia” OR “caudal epidural anesthesia” OR “caudal epidural anaesthesia” OR “caudal nerve block” OR “caudal epidural analgesia” OR “caudal analgesia” OR “sacral block” OR “sacral epidural block” OR “sacral epidural” OR “sacral nerve root block” OR “sacral anesthesia” OR “sacral anaesthesia” OR “sacral analgesia” OR “sacral hiatus epidural injections” OR “caudal epidural” OR CEB OR CB OR CNB)”.

The references from all included studies, previous systematic reviews, and meta-analyses were also manually searched for additional studies. Two authors (D.C. and B.B.M.) extracted baseline characteristics and outcome data from the included studies. When not explicitly mentioned in the text, data was manually extracted from graphics using Web- PlotDigitizer.^13^ Disagreements, such as whether to include conference abstracts, were resolved through consensus and senior author consultation.

### Endpoints

The endpoints analyzed were I) Time to first analgesic request, defined as the interval from block completion to the first administration of postoperative analgesia in response to clinically significant pain, typically a Face, Leg, Activity, Cry, Consolability (FLACC) score ≥ 4; II) FLACC scores at 2 and 24 hours postoperatively; III) Postoperative Nausea and Vomiting (PONV), defined as any reported episode of nausea or vomiting in the postoperative period; and IV) Urinary retention, reported as a binary outcome without standardized diagnostic criteria across studies. A leave-one-out sensitivity analysis was conducted to assess the robustness of our results, as detailed in the Statistical Analysis section. We also contacted the corresponding authors of all included trials to request additional data for subgroup analyses to explore the observed heterogeneity. However, despite our attempts, we could not obtain any responses from the contacted authors.

### Risk of bias and quality assessment

This meta-analysis employed the Cochrane Collaboration tool to assess the risk of bias in randomized trials (Rob-2) and evaluate individual RCTs’ quality.^14^ Two authors (O.R.G. and D.C.) independently conducted the quality assessment. Any disagreements were resolved through senior author consultation (R.A.L.). Trials were individually analyzed for their risk of bias in five domains: randomization process, deviations from intended interventions, missing outcomes, measurement of outcomes, and selection of reported results.^14^ Each RCT was then classified as very low, low, moderate, or high quality of evidence. Grading of Recommendation, Assessment, Development and Evaluations (GRADE) guidelines were applied to inquire about the overall quality of evidence.^15^ Specified data is available in Supplementary Table 2.

### Statistical analysis

Statistical analysis was performed using RStudio version 4.4.0 (2024-04-24). We analyzed binary outcomes by estimating Risk Ratios (RRs) with 95% Confidence Intervals (95% CIs), applying the Mantel-Haenszel method. This approach is used for dichotomous data, with reliable estimates when sample sizes are limited, or events are infrequent. For continuous outcomes, Mean Differences (MDs) were used when studies employed the same measurement scale, while Standardized Mean Differences (SMDs) were applied when scales differed; both were pooled using the inverse variance method, which weights studies by the precision of their estimates accounting for between-study variability.^12^ Heterogeneity was assessed using the Cochrane *Q*-test and the *I^2^* statistic, which quantifies the proportion of variation due to heterogeneity rather than chance.^12^ An *I^2^* value greater than 50% was considered indicative of substantial heterogeneity.^12^

Given the diversity in patient populations, surgical procedures, and outcome definitions across studies, we anticipated moderate-to-high heterogeneity and therefore selected a random-effects model from the outset. To examine the stability of our findings, we performed a leave-one-out analysis, systematically removing one study at a time and recalculating the pooled estimates to identify any single study that might disproportionately influence the results. All analyses were conducted using restricted maximum-likelihood estimators.

In cases where data were reported as medians with interquartile ranges, we estimated means and standard deviations using the approach described by Wan and Luo.^16^^,^^17^ When these converted values suggested skewed distributions, the corresponding outcomes were excluded from the meta-analysis. As fewer than 10 studies were available for most comparisons, we did not perform formal tests for publication bias, in line with recommendations from the Cochrane Handbook.^11^^,^^18^

## Results

### Study selection and baseline characteristics

As detailed in Supplementary Figure 1, the initial search yielded 166 results. We removed duplicated records and ineligible studies, leaving 84. Of these, we excluded 60 studies based on title and abstract screening for not meeting the inclusion criteria. We then assessed the full texts of 24 studies and included 9 Randomized Controlled Trials (RCTs) after excluding observational studies, conference abstracts, and ongoing trials.^2^, ^3^, ^4^^,^^19^, ^20^, ^21^, ^22^, ^23^, ^24^ In total, 507 pediatric patients were analyzed: 248 received ESPB and 259 received CEB. Most patients were younger than 10-years-old, and among studies reporting gender, the majority were male, approximately 68%, although several trials did not specify gender distribution. The included studies did not enroll premature infants or low birth weight populations. We contacted the corresponding authors of all included RCTs to obtain stratified data by age group and surgical type; however, no responses were received. Baseline patient characteristics are summarized in Supplementary Table 3.

### Quality and evidence assessment

Two reviewers (O.R.G. and D.C.) independently evaluated the risk of bias for each included randomized controlled trial using the Cochrane Risk of Bias tool, version 2 (RoB-2).^14^ Among the nine studies, seven were deemed low risk of bias, while two had “some concerns” due to deviations from the intended interventions (Supplementary Table 2).

The certainty of evidence was assessed using the GRADE framework.^15^ For time-to-first rescue analgesia, the quality of evidence was rated as very low, primarily due to significant heterogeneity and imprecision. Evidence supporting the use of ESPB for early postoperative pain relief, reflected by FLACC scores at 2 hours, was graded as high certainty. In contrast, the evidence for FLACC scores at 24 hours was downgraded to moderate because of inconsistencies between study results. High-certainty ratings were also assigned to the outcomes of urinary retention and PONV; however, for PONV, the confidence interval did not indicate a meaningful difference between groups (Supplementary Table 1).

Following Cochrane Handbook guidance (18,23), a formal assessment of publication bias was not performed, as fewer than ten studies were included in the meta-analysis, limiting the validity of such tests.

#### Pooled analysis of all studies

Three studies reported FLACC scores at 2 and 24 hours. At 2 hours, the CEB group showed a non-significant reduction compared to ESPB (MD = -0.11; 95% CI -0.38 to 0.15; p = 0.41; *I^2^* = 0%; 182 patients; Supplementary Fig. 2). At 24 hours, the CEB group continued to show lower scores without reaching statistical significance (MD = -0.30; 95% CI -0.92 to 0.33; p = 0.19; *I^2^* = 41%; 130 patients; Supplementary Fig. 3).

We found no statistically significant difference in time to the first analgesic request between groups (MD = 3.71 min; 95% CI -1.88 to 9.29 min; p = 0.19; *I*^2^ = 99%; 4 studies; 264 patients; Supplementary Fig. 4). The ESPB group had a statistically significantly decreased incidence of urinary retention (RR = 0.12; 95% CI 0.02 to 0.94; p = 0.04; *I*^2^ = 0%; 3 studies; 152 patients; Supplementary Fig. 5). Three studies reported no cases of PONV in either group. One RCT reported low rates in both groups with no statistical difference (RR = 1.14; 95% CI 0.78 to 1.68; p = 0.50; 4 studies; 225 patients; Supplementary Fig. 6).

We performed leave-one-out sensitivity analyses to explore sources of heterogeneity (Supplementary Figs. 7, 8, 9, 10, 11, 12). These analyses did not reduce heterogeneity. We also applied GRADE, RoB-2, standardized outcome definitions, and consistent statistical methods. We contacted study authors to obtain subgroup data, but did not receive responses.

## Discussion

In this systematic review with a meta-analysis of nine RCTs encompassing 507 patients, we compared ESPB versus CEB in pediatric patients undergoing abdominal and sub-abdominal surgeries.^2^, ^3^, ^4^^,^^19^, ^20^, ^21^, ^22^, ^23^, ^24^ Our main findings are: I) No statistically significant difference in the time to first analgesic request between groups; II) No statistically significant difference in FLACC scores at 2 and 24 hours postoperatively between groups; III) A statistically significant decrease in the incidence of urinary retention in the ESPB group; and IV) No episodes of PONV reported in either group across three studies, while the only study contributing to analyzable data showed no significant difference between groups.

The lack of statistically significant differences in the time to first rescue analgesia and FLACC scores at 2 and 24 hours can be explained by the comparable duration of analgesia provided by ESPB and CEB. Two previous meta-analyses have reported that ESPB provides analgesia lasting approximately 6- to 12-hours, with the effect tapering off by 12 hours.^1^^,^^25^ In comparison, Beyaz et al. found that single-shot CEB with levobupivacaine lasts approximately 6 hours, and Wiegele et al. noted that it can extend up to 24 hours by adding adjuvants. These overlapping durations likely resulted in similar early postoperative pain control, explaining the comparable FLACC scores at 2 hours. By 24 hours, both blocks would have largely worn off in most patients, further reducing any observable differences. The alignment of these temporal analgesic profiles likely accounts for the lack of statistically significant findings across these outcomes.

The statistically significant decrease in urinary retention in the ESPB group can be accounted for by the disruption by caudal anesthesia of the parasympathetic outflow and afferent bladder signaling at the sacral level, resulting in diminished detrusor contractility and decreased bladder fullness sensation, mechanisms not impacted by ESPB.^26^^,^^27^ Urinary retention is a recognized complication of caudal block, but it is primarily linked to the use of neuraxial opioids such as caudal morphine. The incidence is low in their absence, particularly when no urologic surgery is involved.^28^ These neural and pharmacologic factors likely account for the lower rate of urinary retention observed with ESPB.

Our results share some similarities with those of Luo et al. (2021)^1^ and Park et al. (2024),^29^ two recent meta-analyses that examined ESPB in pediatric surgery. Luo’s review suggested a modest benefit of ESPB over no block in reducing early postoperative pain and the need for rescue analgesia. However, the strength of the evidence was limited by low certainty. Park’s analysis, which included a more significant number of trials and a broader range of comparators, found improved pain control and reduced opioid use with ESPB. However, neither study directly compared ESPB with CEB, a widely used technique in children. By narrowing the comparison to ESPB versus CEB in a pediatric population, our analysis adds a more specific perspective. While we did not find a difference in pain-related outcomes, we observed a lower urinary retention rate in the ESPB group, an endpoint not explored in those reviews. In addition, we applied stricter eligibility criteria, included only RCTs, assessed study quality using GRADE and RoB-2 tools, and conducted sensitivity analyses to evaluate consistency, aiming to provide reliable data that reflect current clinical practice.

This systematic review with meta-analysis indicates that ESPB and CEB are equally effective in managing pain for children undergoing abdominal and sub-abdominal surgeries, as there were no major differences in pain levels or the time it took to receive first pain relief. However, the notable decrease in Postoperative Urinary Retention (POUR) with ESPB is important because POUR can lead to longer hospital stays (7.8 vs. 1.7 days), higher healthcare costs, and a greater risk of infections. Lower POUR rates in the ESPB group could improve patient comfort, reduce the need for catheterization, and lower the incidence of urinary tract infections and related complications.^30^ These advantages may be particularly valuable in outpatient procedures or patients with elevated urinary retention risk, where early discharge and reduced postoperative burden are paramount.

There was high heterogeneity in the time to first rescue analgesia (*I²* = 99%), even though there was a uniform definition across studies. While Bansal et al. (2024)^3^ appeared to contribute notably to the inconsistency based on the Baujat plot, removing this study from the analysis only slightly reduced the heterogeneity (to 97%). The result indicates that the variability likely stems from multiple underlying factors rather than a single influential study. These include differences in surgical procedures, local anesthetic type, concentration, and volume, as well as postoperative analgesia protocols as detailed in Supplementary Table 3. For instance, bupivacaine concentrations ranged from 0.125% to 0.25%, and volumes from 0.16 to 1.2 mL.kg^-1^.

We were unable to perform a meta-regression because of the limited number of studies. In addition, age-associated variation in pain perception may have added variance, but age-stratified data were not available despite efforts to contact the authors. With the present findings, the necessity for standardized protocols and detailed reporting becomes apparent when comparing future trials to limit heterogeneity.

This meta-analysis has several limitations. First, although the overall risk of bias was low in most included studies, two trials were rated as having “some concerns” due to deviations from intended interventions, potentially affecting internal validity. Second, the high heterogeneity observed in our primary outcome, time to first rescue analgesia (*I²* = 99%), limits the interpretation of pooled results. Third, our sample size of 507 patients may be underpowered to detect small but significant differences. Fourth, pain assessments using FLACC scores introduce subjectivity, especially in younger children, which may vary by evaluator and institutional protocols. Finally, since most studies excluded patients with unsuccessful blocks, we have the possibility of selection bias by overestimating the effectiveness of both ESPB and CEB.

## Conclusion

This meta-analysis indicates that ESPB and CEB are similarly effective for pediatric abdominal and sub-abdominal operations, with no difference in early pain scores and time to first request for analgesia. There were fewer instances of urinary retention with ESPB. A cautious interpretation of these findings should be exercised because they were marred by heterogeneity and methodological limitations, but ESPB could be a more favorable choice for those at increased risk of urinary retention.

## Abbreviations

CEB, Caudal Epidural Block; CI, Confidence Interval; e.g., exempli gratia; ESPB, Erector Spinae Plane Block; FLACC, Face, Leg, Activity, Cry, Consolability; GRADE, Grading of Recommendation, Assessment, Development and Evaluations; MD, Mean Difference; PONV, Postoperative Nausea and Vomiting; POUR, Postoperative Urinary Retention; PRISMA, Preferred Reporting Items for Systematic Reviews and Meta-Analysis; RCT, Randomized Controlled Trial; RR, Risk Ratio; SMD, Standardized Mean Difference.

## Statement of ethics

An ethics statement is not applicable because this study is based exclusively on published literature.

## Data availability statement

The authors confirm that the data supports the findings of this study.

## Authors’ contributions

All authors contributed equally to the production of this manuscript.

## Funding

This research received no specific grant from funding agencies in the public, commercial, or not-for-profit sectors.

## Conflicts of interest

All authors report no relationships that could be considered conflicts of interest. All authors take responsibility for all aspects of the reliability and freedom from bias of the data presented and their discussed interpretation.

## Appendix Supplementary materials

Image, application 1

## Footnotes

Supplementary material associated with this article can be found in the online version at doi:10.1016/j.bjane.2025.844640.
