# Supplementary material for: Erector spinae plane block versus caudal epidural block in pediatric surgery: a systematic review and meta-analysis of randomized clinical trials
Source: Braz J Anesthesiol. 2025 May 16;75(4):844640. doi: 10.1016/j.bjane.2025.844640 (PMC12174558; doi:10.1016/j.bjane.2025.844640)

**BJAN-D-24-00528_Supplementary Material**

**Table 1** Quality of evidence: grade assessment scale.

| **Erector Spinae Plane Block compared to Caudal Epidural Block for Pediatric Surgery** | | | | | |
| --- | --- | --- | --- | --- | --- |
| **Patient or population:** Pediatric Surgery | | | | | |
| **Setting:** | | | | | |
| **Intervention:** Erector Spinae Plane Block | | | | | |
| **Comparison:** Caudal Epidural Block | | | | | |
| **Outcomes** | **No of participants (studies) Follow-up** | **Certainty of the evidence (GRADE)** | **Relative effect (95% CI)** | **Anticipated absolute effects** | |
|  |  |  |  | **Risk with caudal epidural block** | **Risk difference with Erector Spinae Plane Block** |
| Time to first rescue analgesia | 264 (4 RCTs) | ⊕⊕⊕⊕ High |  |  | MD **3.71 hours higher** (1.88 lower to 9.29 higher) |
| FLACC scores at 2 hours | 182 (3 RCTs) | ⊕⊕⊕⊕ High |  |  | MD **0.11 hour lower** (0.38 lower to 0.15 higher) |
| FLACC scores at 24 hours | 130 (2 RCTs) | ⊕⊕⊕⊕ High |  |  | MD **0.38 hour lower** (0.92 lower to 0.33 higher) |
| Urinary retention | 152 (3 RCTs) | ⊕⊕⊕⊕ High | **RR** **0.121** (0.016 to 0.943) | 105 per 1,000 | **93 fewer per 1,000** (104 fewer to 6 fewer) |
| Postoperative Nausea and Vomiting and Urinary Retention (PONV) | 48 (3 RCTs) | ⊕⊕⊕⊕ High | **RR** **1.143** (0.735 to 1.776) | 583 per 1.000 | **83 more per 1,000** (155 fewer to 453 more) |
| * **The risk in the intervention group** (and its 95% Confidence Interval) is based on the assumed risk in the comparison group and the relative effect of the intervention (and its 95% CI). CI, Confidence Interval; MD, Mean Difference; RR, Risk Ratio. | | | | | |
| **GRADE Working Group grades of evidence:** | | | | | |
| High certainty: We are very confident that the true effect lies close to that of the estimate of the effect. | | | | | |
| Moderate certainty: We are moderately confident in the effect estimate: the true effect is likely to be close to the estimate of the effect, but there is a possibility that it is substantially different. Low certainty: our confidence in the effect estimate is limited: the true effect may be substantially different from the estimate of the effect. | | | | | |
| Very low certainty: We have very little confidence in the effect estimate: the true effect is likely to be substantially different from the estimate of effect. | | | | | |

**Table 2** Critical appraisal of individual studies according to the Cochrane Collaboration’s tool for assessing risk of bias in randomized trials.

| **Study** | **Bias from randomization process** | **Bias due to deviations from intended interventions** | **Bias due to missing outcome data** | **Bias in measurement of the outcomes** | **Bias in selection of the reported result** | **Overall risk of bias** |
| --- | --- | --- | --- | --- | --- | --- |
| Abdelrazik 2022 | Low | Low | Low | Low | Low | Low |
| Abotaleb 2023 | Low | Low | Low | Low | Low | Low |
| Bansal 2024 | Low | Low | Low | Low | Low | Low |
| Elbadry 2023 | Low | Some concerns | Low | Low | Low | Some concerns |
| Elshazly 2023 | Low | Some concerns | Low | Low | Low | Some concerns |
| Guan 2023 | Low | Low | Low | Low | Low | Low |
| Mandour 2023 | Low | Low | Low | Low | Low |  |
| Ozen 2023 |  |  |  |  |  |  |
| Pandey 2024 | Low | Low | Low | Low | Low | Low |

^12^Sterne JAC, Savović J, Page MJ, Elbers RG, Blencowe NS, Boutron I, Cates CJ, Cheng H-Y, Corbett MS, Eldridge SM, Hernán MA, Hopewell S, Hróbjartsson A, Junqueira DR, Jüni P, Kirkham JJ, Lasserson T, Li T, McAleenan A, Reeves BC, Shepperd S, Shrier I, Stewart LA, Tilling K, White IR, Whiting PF, Higgins JPT. RoB2: a revised tool for assessing risk of bias in randomized trials. BMJ. 2019;366:l4898.

**Table 3** Baseline characteristics of included studies.

| **Study** | **Design** | **Patients ESPB / CEB** | **Male (%) ESPB / CEB** | **Age (years)^a^ ESPB / CEB** | **ASA I ESPB / CEB** | **ASA II ESPB\|/CEB** | **Weight (kg)^k^ ESPB / CEB** | **Type of Surgery ESPB/CEB** | **Surgical Duration (minutes)^m^ ESPB / CB** | **Anesthesia duration (minutes)^m^ ESPB / CB** | **LA* ESPB/CEB** | **LA* Dosage^z^ ESPB /CEB (mL.kg^-1^)** |
| --- | --- | --- | --- | --- | --- | --- | --- | --- | --- | --- | --- | --- |
| Abdelrazik 2022[4] | RCT | 20/20 | 8 (40%) / 10 (50%) | 7.66±3.49 | NA | NA | 19.05±4.95 / 17.20±5.72 | Inguinal herniotomy | 28.3±3.4 / 27.6±4.5 | 47.5±5.4 / 48.7±4.7 | Bupivacaine 0.25% | 0.16/1 |
| Abotaleb 2023[2] | RCT | 25/25 | 12 (48%) / 15 (60%) | 7.96±3.55 / 7.36±3.49 | 21 (84%) / 19 (76%) | 4 (16%) / 6 (24%) | 27.8±11.52 / 26.2±11.02 | Fracture shaft femur / Fracture shaft tibia / Genu varum: High tibial osteotomy, or Distal femur osteotomy. | 134.4±26.43 / 138.2±25.57 | 153.6±25.72 / 158.2±27.57 | Bupivacaine 0.25% | 0.5/0.5 |
| Bansal 2024[1] | RCT | 25/25 | 25 (100%) / 25 (100%) | NA | NA | NA | 20.68±4.96 / 19.84±4.31 | NA | 57.0±3.23 / 55.8±4.25 | NA | Bupivacaine 0.25% | 1/0.5 |
| Elbadry 2023[9] | RCT | 29/40 | NA | 2.85±1.29 / 2.75±1.35 | 37 (94.87%) / 38 (95%) | 2 (5.13%) / 2(5%) | 14.82 ±2.33 / 14.93 ± 2.37 | NA | 118.08±35.59 / 115.5±3.36 | NA | Bupivacaine 0.25% | 1/1 |
| Elshazly 2023[8] | RCT | 30/30 | NA | 4.57±2.99 / 4.16±3.76 | 38 (100%) / 37 (97.4%) | 0 (0.0) / 1 (2.6) | 18.92±8.77 / 18.58±9.78 | Hip or proximal femur fracture; Developmental dysplasia of the hip; Slipped capital femoral epiphysis; Developmental coxa vera | 61.95±17.96 / 78.08±24.11^b^ | 79.74±8.67 / 92.05±25.12^b^ | Bupivacaine 0.25% | 0.5/0.5 |
| Guan 2023[3] | RCT | 38/38 | 12 (48%) / 15 (60%) | 7.96±3.5 / 7.36 ±3.49 | 21 (84%) / 19 (76%) | 4 (16%) / 6 (24%) | 27.8±11.5 / 26.2 ±11.02 | NA | 134.4±26.43 / 138.2±25.57 | 153.6±25.72 / 158.2±27.57 | Ropivacaine 0.2% | 0.5/1 |
| Mandour 2023[5] | RCT | 25/25 | NA | NA | 24 (96%) / 23 (92%) | 1 (4%) / 2 (8%) | 17.40±3.25 / 17.20±3.55 | Nephrectomy / Pyeloplasty | 93±19.2 / 88.1±19 | NA | Bupivacaine 0.125% | 0.5/1.2 |
| Özen 2023[6] | RCT | 30/30 | 60 (100%) | 4±1.51/4.36 | NA | NA | 16.14±5.06 / 16.35±5.84 | Circumcision + inguinal hernia / Circumcision + hydrocelectomy / Circumcision + orchiopexy / Circumcision + orchiopexy | 37.73±5.64 / 39.50±2.34 | NA | Bupivacaine 0.25% | 0.5/0.5 |
| Pandey 2024[7] | RCT | 26/26 | 20 (76.9%) / 21 (80.8%) | 4.64±2.353/4.14±3.14 | 23 (88.4%) / 24 (92.3%) | 3(11.5%)/2 (7.7%) | 14.78±3.6 / 15.89±7.76 | Inguinal herniotomy; Orchidopexy; Pyeloplasty | 2±1.57 / 1.64±0.7843^a^ | NA | Bupivacaine 0.25% | 0.5/1 |

^a^ Expressed in years; ^k^ Expressed in kilograms; ^m^ Expressed in minutes; ^h^ Expressed in hours; NA, Not Available Data; RCT, Randomized Clinical Trial; ASA, American Society of Anesthesiologists; CB, Caudal Block; ESPB, Erector Spinae Plane Block; ^b^LA, Local Anesthetic; ^z^ Expressed in mL.kg^-1^.

**Figure 1** PRISMA flow diagram of study screening and selection: the process of the study selection is explained here, showing the number of studies identified, screened, assessed for eligibility, and ultimately included in the review, with reasons for exclusions at each stage.


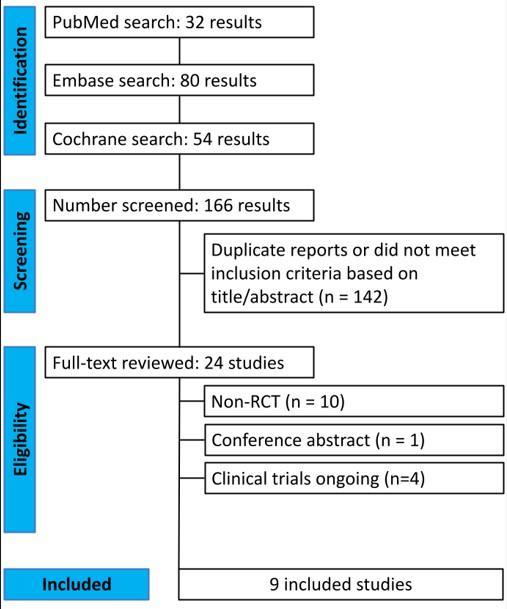


**Figure 2** Leave-one-out analysis for time to first rescue analgesia: when a specific study is removed from the analysis, the result would be as described.


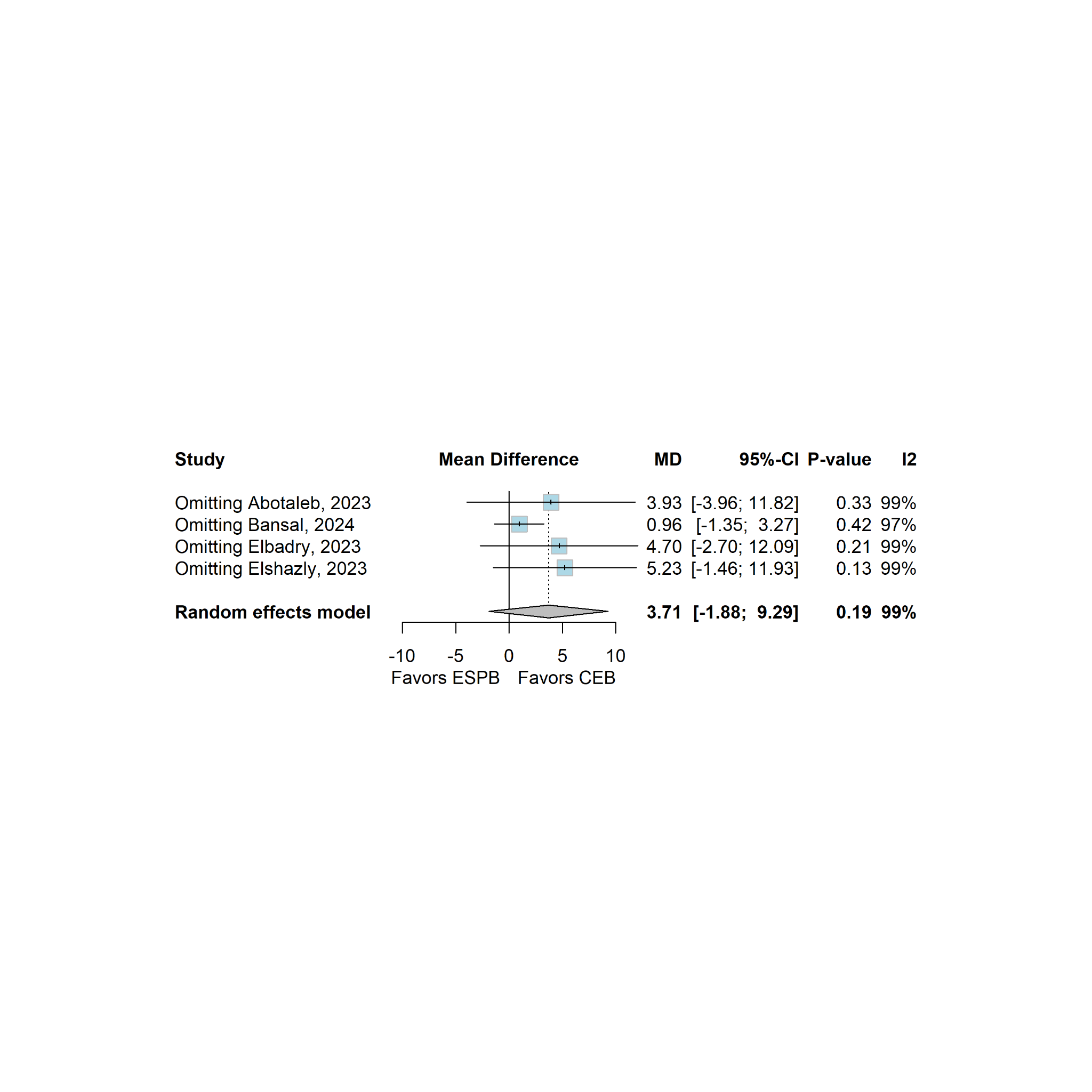


**Figure 3** Leave-one-out analysis for FLACC at 2 hours: when a specific study is removed from the analysis, the result would be as described.


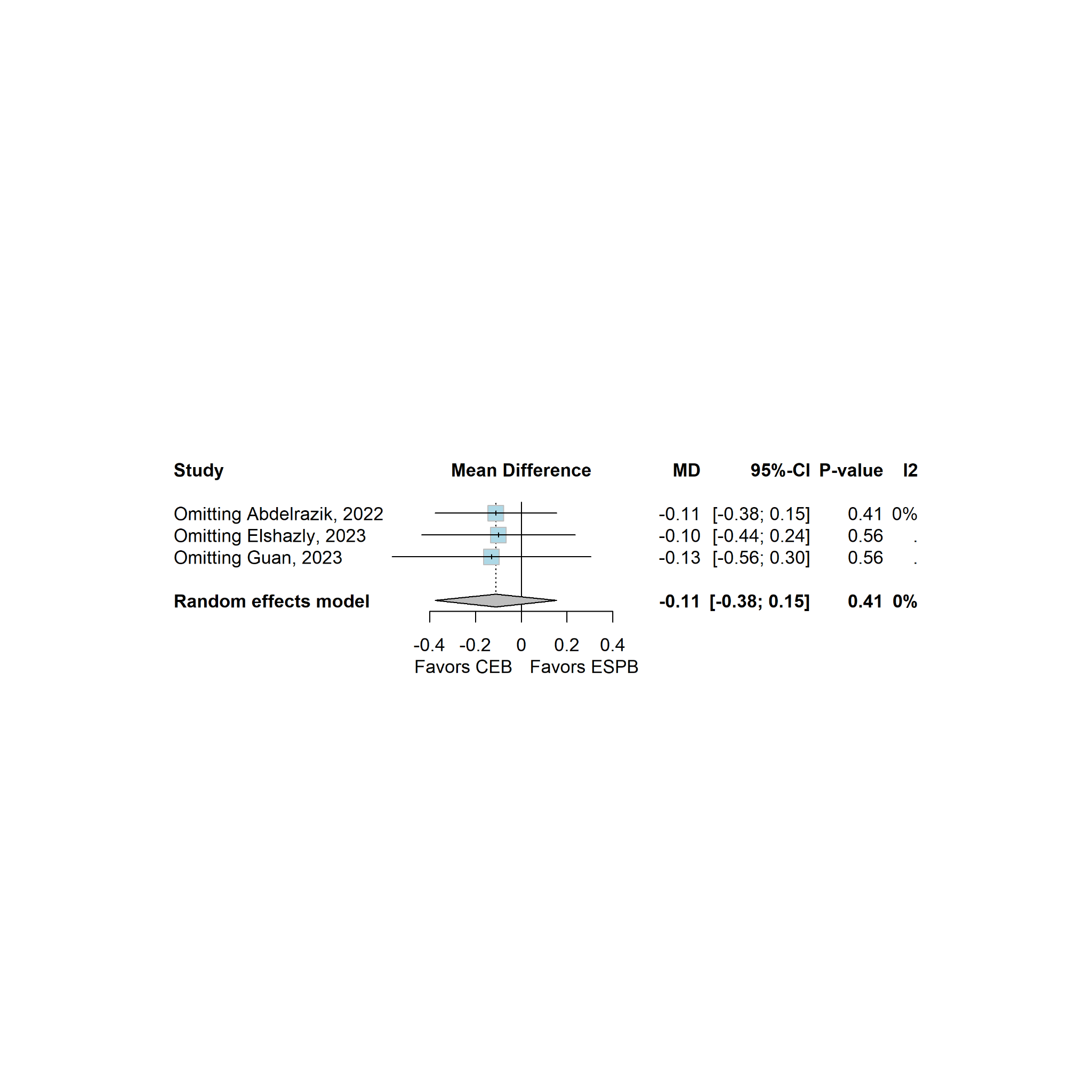


**Figure 4** Baujat plot for time to first analgesic request demonstrating studies that most contributed to heterogeneity.


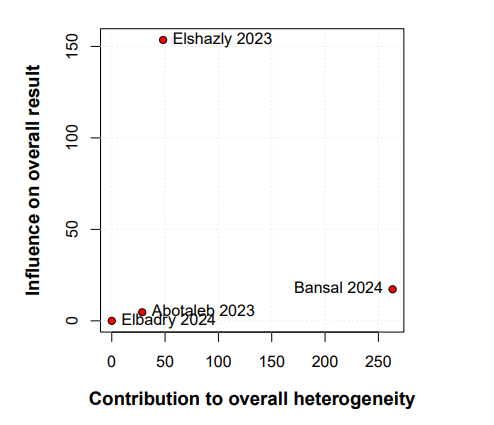


**Figure 5** Leave-one-out analysis for postoperative nausea and vomiting: when a specific study is removed from the analysis, the result would be as described.


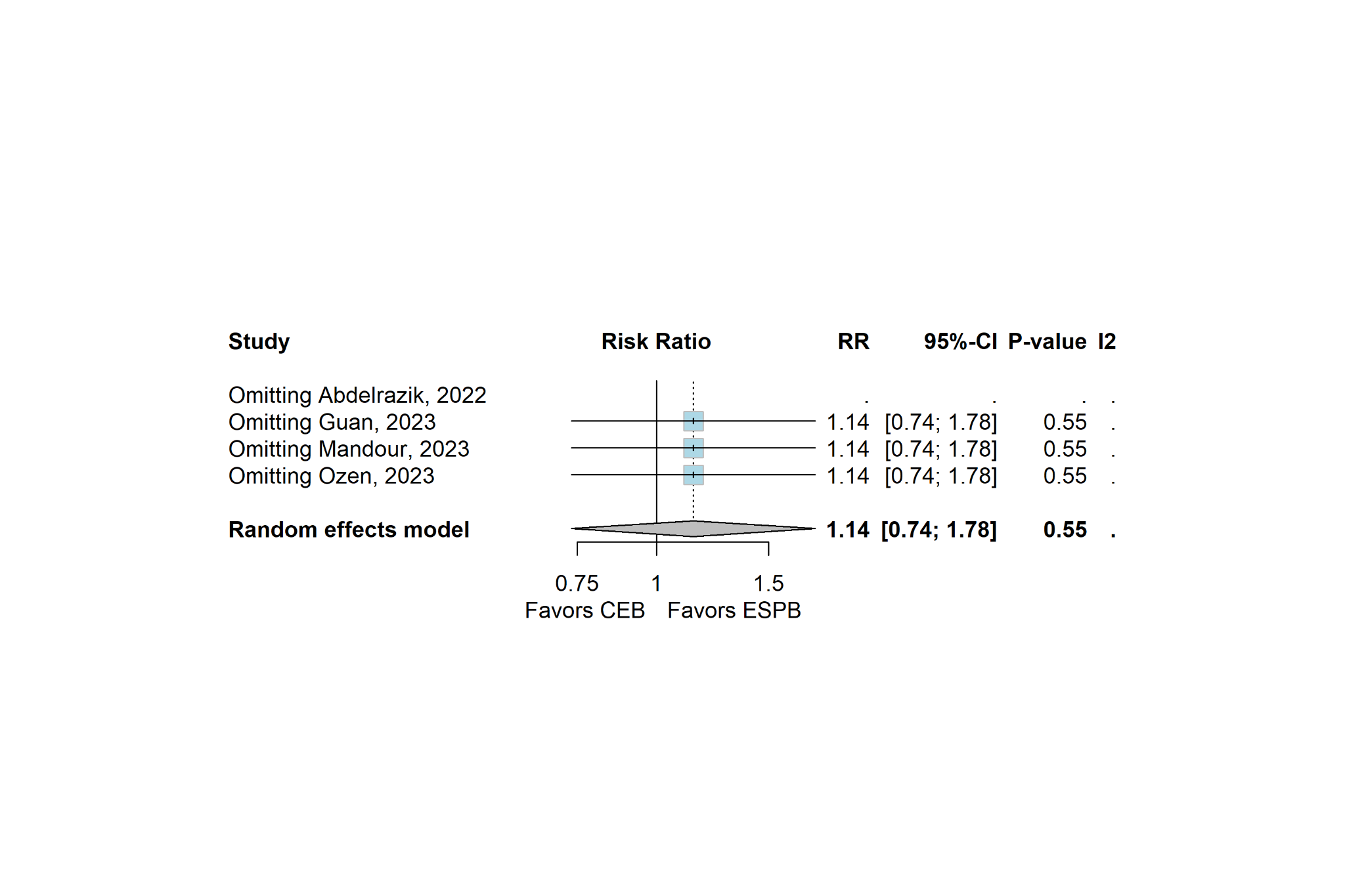


**Figure 6** Leave-one-out analysis for FLACC at 24 hours: when a specific study is removed from the analysis, the result would be as described.


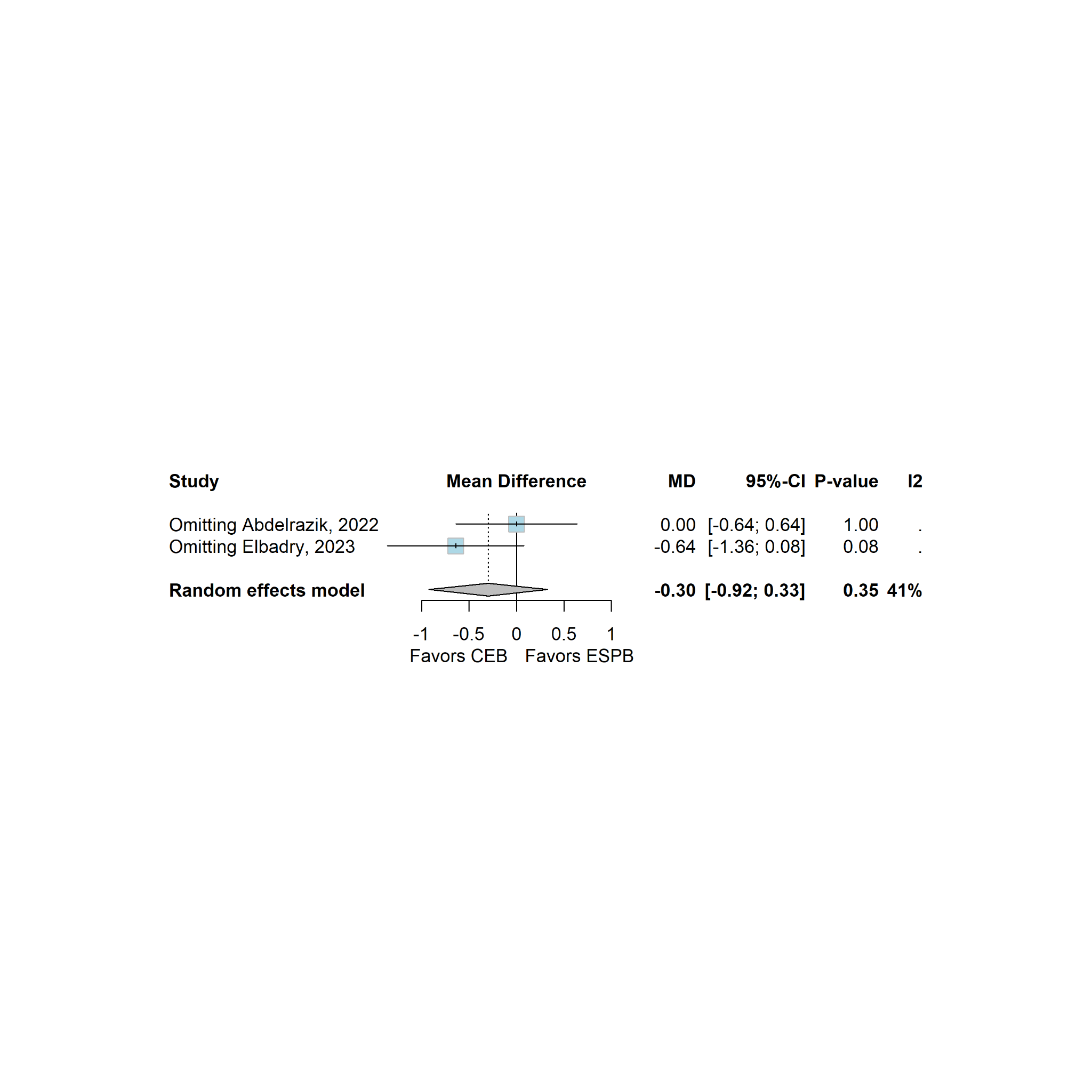


**Figure 7** Forest plot for time to first rescue analgesia demonstrating high heterogeneity across studies and no statistically significant difference between groups.


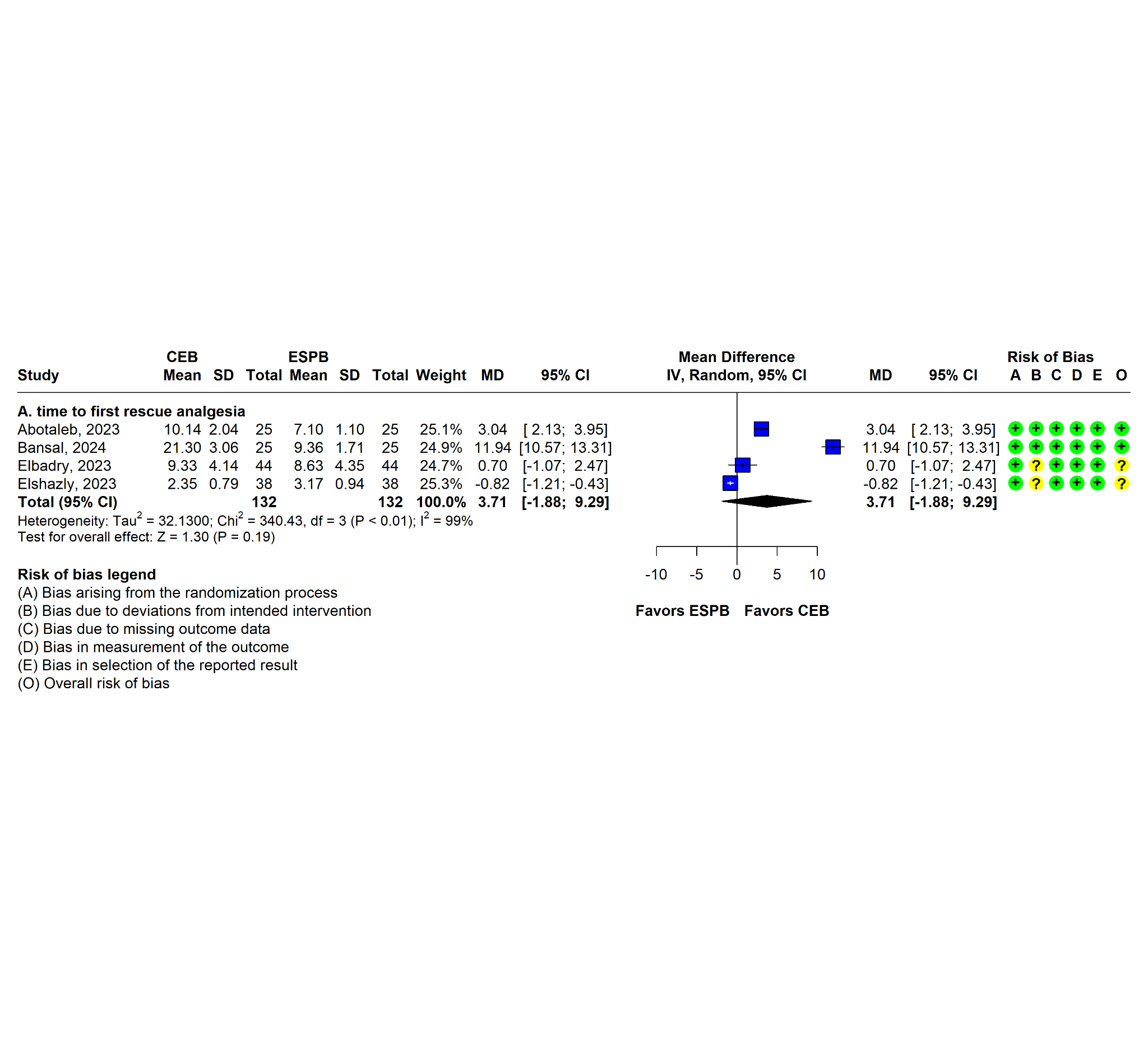


**Figure 8** Forest plot for FLACC score at 2h indicating superior early postoperative pain control with ESPB.


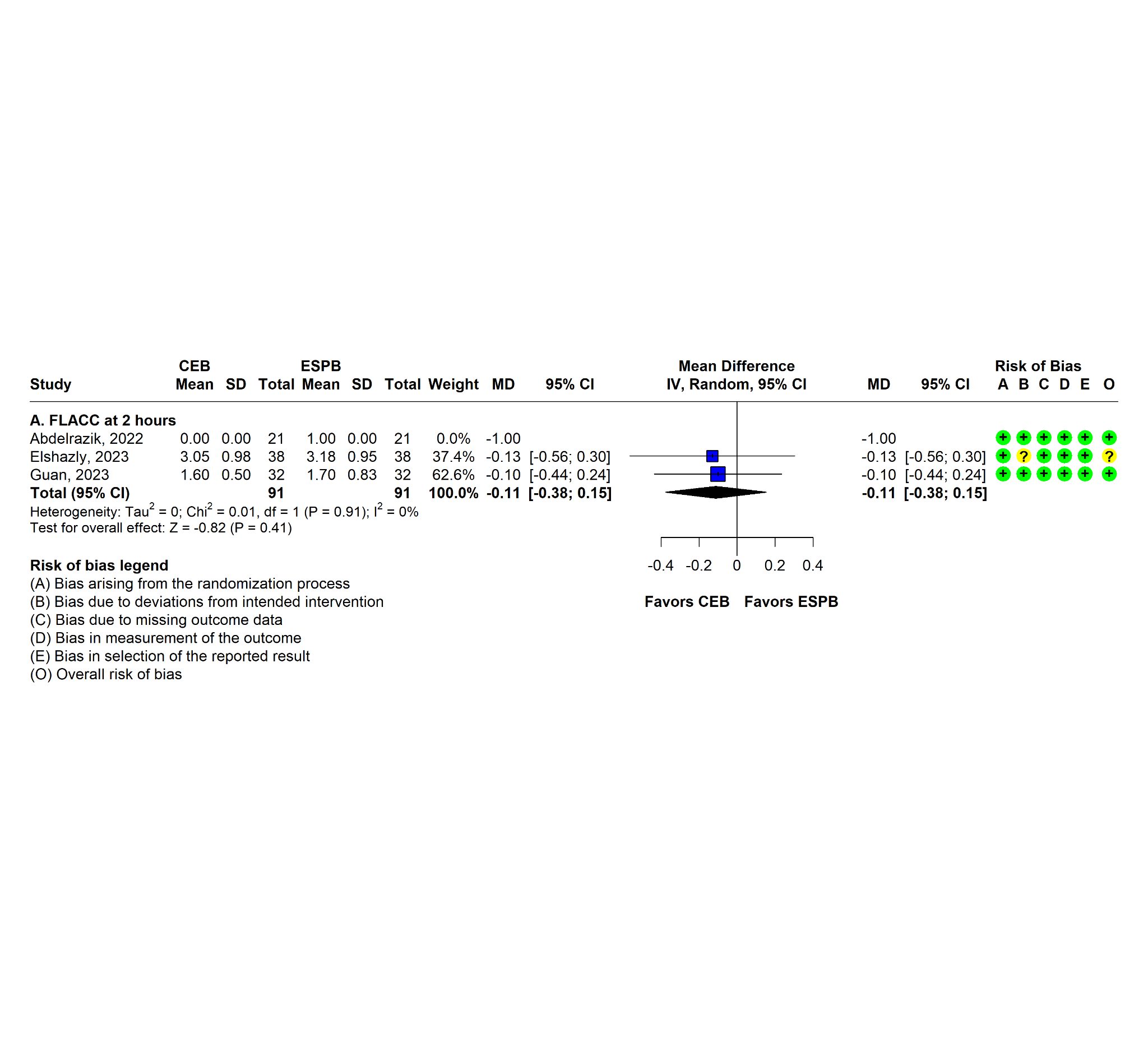


**Figure 9** Forest plot for FLACC score at 24h indicating moderate heterogeneity across studies.


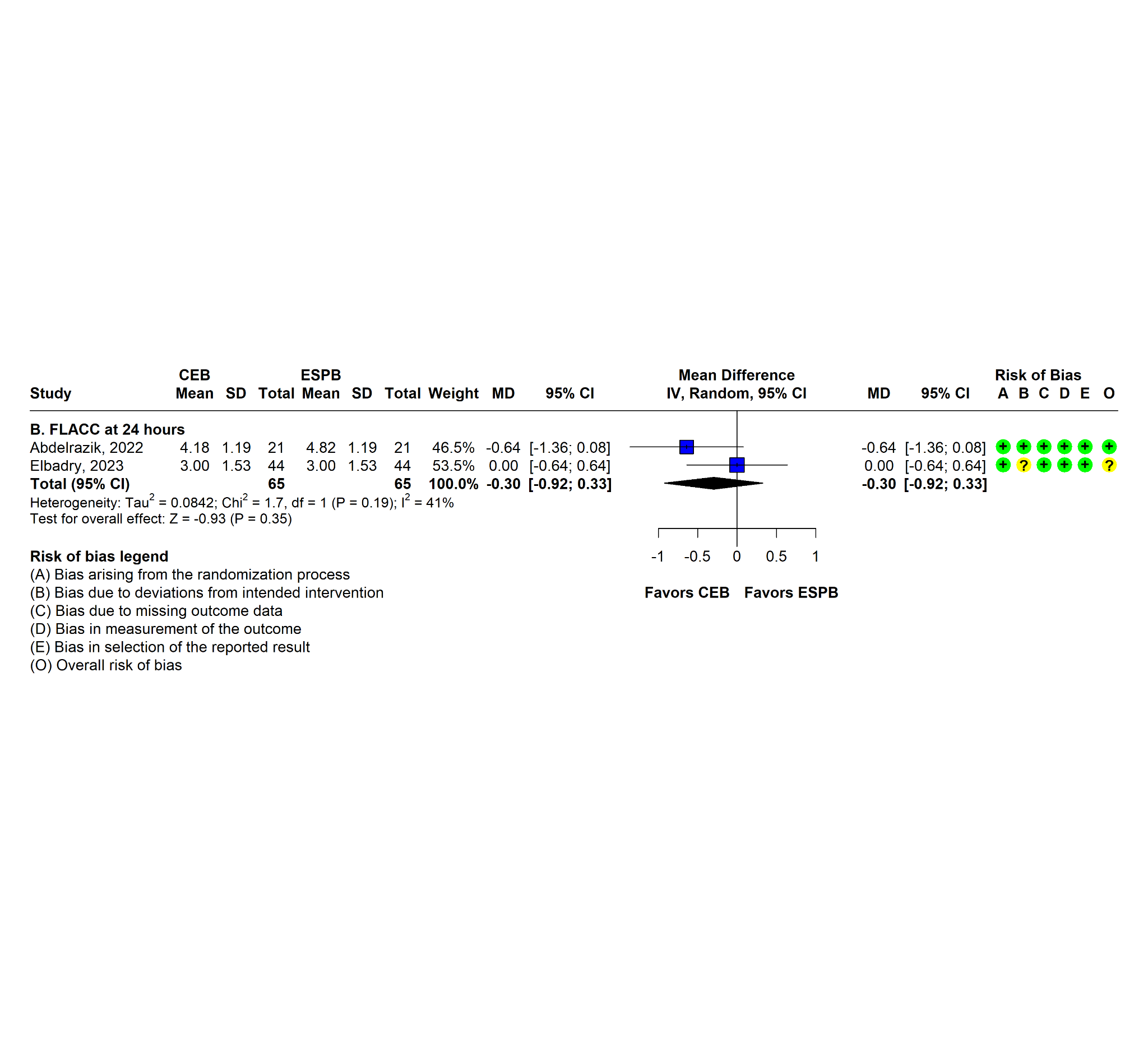


**Figure 10** Forest plot for postoperative nausea and vomiting demonstrating null results in three studies.


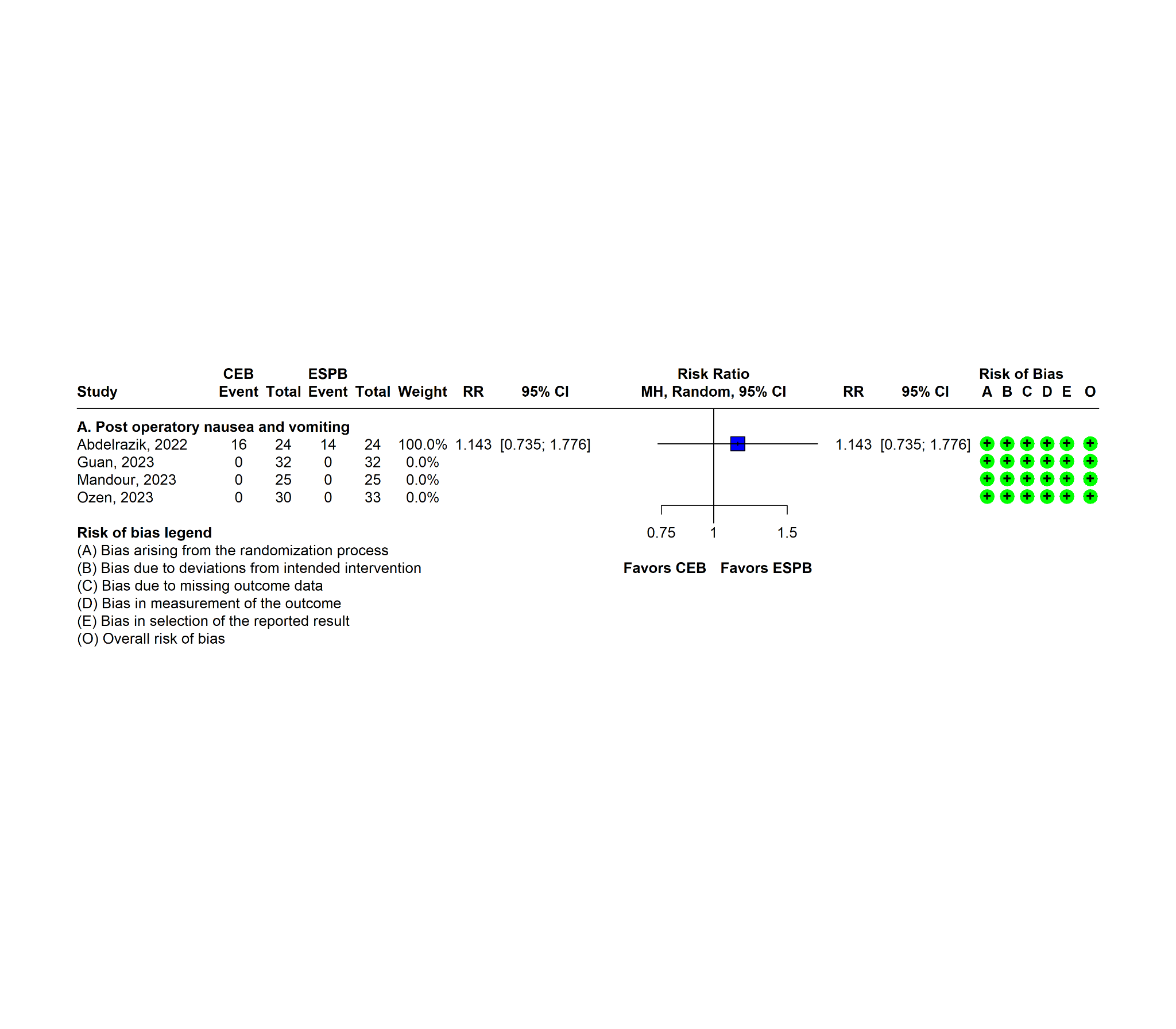


**Figure 11** Forest plot for urinary retention demonstrating favoring results for ESPB.


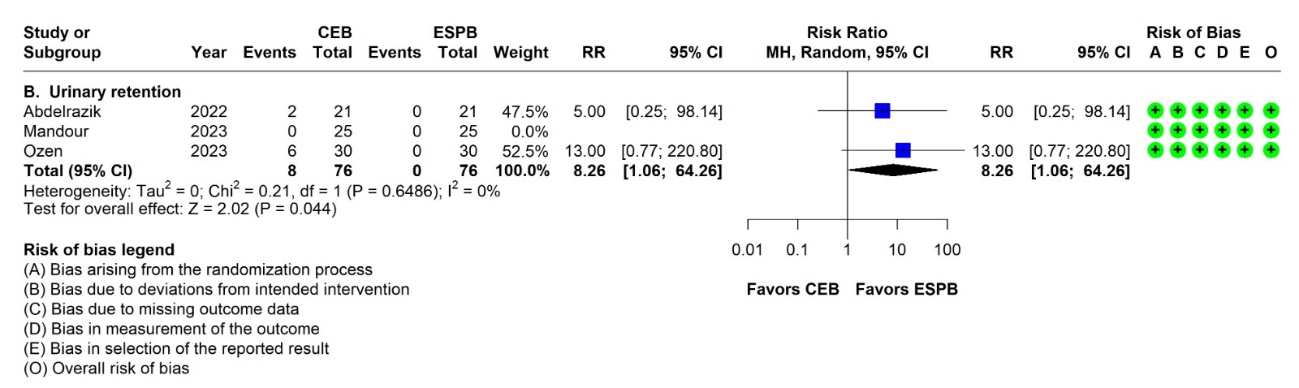


**Figure 12** Leave-one-out analysis for urinary retention: when a specific study is removed from the analysis, the result would be as described.


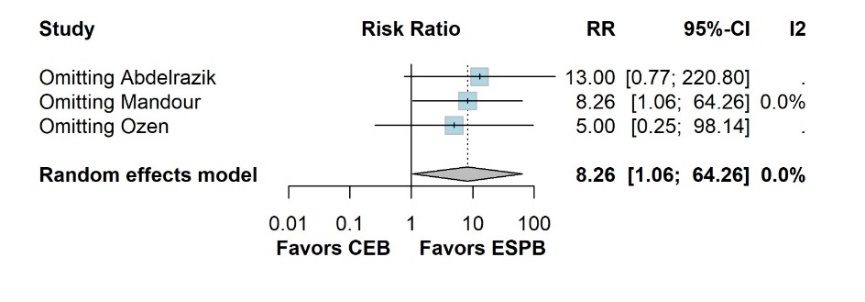

Supplement: Supplementary file 1 [file mmc1.docx]
